# ELTD1 is present in extracellular vesicles derived from endothelial cells as a cleaved extracellular domain which induces in vivo angiogenesis

**DOI:** 10.1002/jex2.52

**Published:** 2022-08-02

**Authors:** Helen Sheldon, Wei Zhang, Esther Bridges, Koon Hwee Ang, Salwa Lin, Massimo Masiero, Demin Li, Penny A. Handford, Pat Whiteman, Roman Fischer, Francesca Buffa, Manu Vatish, Alison H. Banham, Adrian L. Harris

**Affiliations:** ^1^ Cancer Research UK Molecular Oncology Laboratories Weatherall Institute of Molecular Medicine University of Oxford John Radcliffe Hospital Oxford UK; ^2^ Nuffield Department of Women's & Reproductive Health, Women's Centre University of Oxford John Radcliffe Hospital Oxford UK; ^3^ Nuffield Division of Clinical Laboratory Sciences Radcliffe Department of Medicine John Radcliffe Hospital Oxford UK; ^4^ Department of Biochemistry University of Oxford Oxford UK; ^5^ Nuffield Department of Medicine Target Discovery Institute Oxford University, NDM Research Building Oxford UK; ^6^ Department of Oncology University of Oxford Churchill Hospital Oxford UK

**Keywords:** angiogenesis, ELTD1, extracellular vesicles, migrasomes

## Abstract

ELTD1/ADGRL4 is an adhesion GPCR with an important role in angiogenesis. We recently identified a role for ELTD1 in wound repair and inflammation. Activation of ELTD1 in endothelial cells results in a type II EMT to myofibroblast‐like cells that have enhanced angiogenic ability. Furthermore, expression of Eltd1 in murine breast cancer cells increases tumour growth by increasing blood vessel size and perfusion and by creating an immunosuppressive microenvironment. As extracellular vesicles (EVs) are known to be involved in vascular development, growth and maturation we investigated the composition and functional effects of the EVs isolated from ELTD1 expressing cells to elucidate their role in these processes. A highly glycosylated form of the extracellular domain (ECD) of ELTD1 is readily incorporated into EVs. Using mass spectrometry‐based proteomics we identified proteins that are enriched in ELTD1‐EVs and are involved in haemostasis and immune responses. ELTD1 enriched EVs were pro‐angiogenic in vivo and in vitro and the presence of the ECD alone induced endothelial sprouting. In endothelial cells experiencing laminar flow, ELTD1 levels were reduced in the EVs when they are quiescent, showing a relationship between ELTD1 and the activation state of the endothelium. Using FACS, we detected a significant increase in vesicular ELTD1 in the plasma of patients with preeclampsia, a condition characterized by endothelial dysfunction. These data confirm a role for ELTD1 in wound repair and inflammation and reveal its potential as a biomarker of vessel dysfunction.

## 1 INTRODUCTION

Epidermal growth factor, latrophilin and seven‐transmembrane domain‐containing 1 (ELTD1), is an orphan G‐protein‐coupled receptor (GPCR) belonging to the adhesion GPCR (aGPCR) family. It has been assigned to the Latrophilin family and recently re‐designated Adhesion G Protein‐Coupled Receptor L4 (ADGRL4). ELTD1 is composed of an extracellular domain (ECD) with epidermal growth factor (EGF)‐like repeats and a seven‐transmembrane domain (TMD7) followed by a short cytoplasmic tail (Nechiporuk et al., 2001). The extracellular region of ELTD1 also contains a membrane proximal GPCR‐Autoproteolysis Inducing (GAIN) domain which undergoes autocatalytic processing at a GPCR‐proteolytic site (GPS) to give rise to an ECD that is non‐covalently bound at the cell surface (Araç et al., 2012; Lin et al., 2010). Detachment of the ECD leads to activation of receptor signalling in a number of aGPCRs (Okajima et al., 2010; Paavola et al., 2011; Ward et al., 2011).

ELTD1 is expressed in endothelial cells, smooth muscle cells, cardiomyocytes and certain tumour cells (Amour et al., 1998; Herbert et al., 2008; Kan et al., 2018; Nechiporuk et al., 2001; Wallgard et al., 2008). It has an important role in angiogenesis (Masiero et al., 2013; Serban et al., 2015) and higher ELTD1 expression is associated with the vasculature of a number of tumour types and resistance to anti‐angiogenic therapy (Kan et al., 2018; Masiero et al., 2013; Niinivirta et al., 2020). ELTD1 siRNA and anti‐ELTD1 antibodies reduced vessel density in ovarian and glioma tumour models thereby confirming its potential as an anti‐angiogenic therapeutic target (Masiero et al., 2013; Zalles et al., 2020).

We have recently identified a role for ELTD1 in wound repair and inflammation processes (Sheldon, Alexander, et al., 2021; Sheldon, Bridges, et al., 2021). Activation of ELTD1 in endothelial cells results in a type II EMT (EndMT) creating myofibroblast‐like cells which have increased angiogenic ability and secrete chemokines and cytokines that are capable of regulating the immune response (Sheldon et al., 2021). Furthermore, expression of Eltd1 in murine breast cancer tumour cells increased tumour growth and their metastatic potential in syngeneic mouse models. The Eltd1‐expressing tumours had larger, better perfused vessels and they released pro‐angiogenic and immune‐modulating factors that created an immunosuppressive microenvironment (Sheldon et al., 2021).

Extracellular vesicles (EVs) are secreted factors that can also influence angiogenesis, inflammation, tumour growth and wound healing (Kuriyama et al., 2020; Oggero et al., 2019; Zaborowski et al., 2015). They can be broadly grouped into four types; exosomes, microvesicles, apoptotic vesicles and migrasomes and they have different origins, size and function (Doyle & Wang, 2019; Ma et al., 2015). They have gained significant interest in recent years as mediators of intercellular communication through the transfer of proteins, RNA and DNA and they have potential as biomarkers as the composition of EVs reflects that of the cell from which they are derived and they are abundant in many biological fluids (Ciferri et al., 2021). They have a much less complex proteome than cells and changes in EVs protein levels, RNA levels or the appearance of mutated proteins are being explored as biomarkers for a variety of diseases.

We therefore investigated the composition of EVs derived from ELTD1 overexpressing endothelial cells and assessed their functional effects on angiogenesis and tumour growth in vitro and in vivo. FACs suitable antibodies were also generated to detect ELTD1‐positive EVs in plasma to enable us to explore its potential as a biomarker for vessel dysfunction.

## 2 MATERIALS AND METHODS

### 2.1 Cell culture and reagents

Human umbilical cord endothelial cells (HUVEC) and human microvascular cells (HMEC‐1) were purchased from Lonza and cultured in their EGM2 and EGM2‐MV media respectively. MDA‐MB‐231 (ATCC) were cultured in DMEM supplemented with 10% FBS. FGF (R&D Systems) and TNFα (ThermoFisher) were added to cells at 10 ng/ml. Codon optimised ELTD1 or HA‐tagged ELTD1 was cloned into pLenti6.2V5DEST (ThermoFisher) and the empty vector was used as a control. HA‐tagging of ELTD1 did not affect processing, glycosylation or localisation of ELTD1 (Sheldon et al., 2021). Virus was produced in 293T and concentrated by ultracentrifugation using standard techniques. The viruses were titred using blasticidin resistance and endothelial cells infected at MOI 5 for all experiments.

### 2.2 Migration assay

Migration was assessed using the IncuCyte® live cell analysis system (Sartorius). Endothelial cells were grown to confluence in a 24‐well ImageLock plate and a scratch wound was made using the IncuCyte® wound maker (Sartorius). Images were collected every hour until the wound had closed and the images were analysed using Fiji software.

### 2.3 Proliferation assay

HUVEC proliferation was assessed over a 72 h time period, after seeding 1000 cells in a 96‐well plate, using the CYQUANT cell proliferation assay (ThermoFisher) according to manufacturer's instructions.

### 2.4 Endothelial sprouting assay

Endothelial cells were either coated onto Cytodex beads (Sigma) or made into spheroids using the hanging drop technique as previously described (Oon et al., 2017). Cells were then embedded in 2.5 mg/ml Fibrin (Sigma) and images acquired every 24 h using an AMG Evos XL Core digital microscope (Fisher Scientific). The sprouting area was quantified using Fiji software.

#### 2.4.1 Live cell imaging

HUVEC were infected with HA‐tagged ELTD1 as previously described (Sheldon et al., 2021). The cells were then stained with Alexa Fluor 488 HA‐Tag (Cell Signalling) and confocal microscopy performed using an LSM 880 Confocal Microscope (Zeiss).

#### 2.4.2 Application of shear stress

HUVEC (7.5×10^4^) were seeded onto 0.4 mm microslides (μ‐Slide I Luer, ibidi) and used when they were fully confluent. Unidirectional flow of 15 dyn/cm^2^ was applied for 48 h using an ibid pump system housed in a 37°C tissue culture incubator.

### 2.5 Western blotting

Proteins were separated by SDS‐PAGE using standard techniques. Antibodies were purchased from the following companies: ELTD1 (Sigma); β‐actin conjugated to HRP (Sigma), CD63 and CD9 (BD Biosciences), GM130 and Syntenin (Abcam); ITIH4 (Proteintech); ITGA5 (Cell Signalling). Secondary antibodies were purchased from DAKO.

### 2.6 ELISA

Three batches of control and ELTD1 enriched HMEC‐1 EVs were lysed in lysis buffer (CST) and 75μg of total protein each used in triplicate. The Fibrinogen ELISA was performed as per manufacturer's instructions (Abcam)

### 2.7 Angiogenesis array

HUVEC (5 × 10^5^) were incubated for 24 h with 1 × 10^9^/ml EVs (control and ELTD1 expressing) and then lysed in Lysis Buffer 17 (R&D Sysytems). Total 300 μg of each was used in the ProteomeProfiler Angiogenesis Array (R&D Systems) according to manufacturer's instructions.

### 2.8 Antibody production

All in vivo work was approved by local ethics review committee and governed by the appropriate Home Office project (30/3133) and personal licenses. Hybridoma cell lines secreting anti‐ELTD1 mouse monoclonal antibodies were generated by immunising mice with purified bacterially expressed and refolded N‐terminal human ELTD1 EGF domains, as previously described (Köhler & Milstein, 1975). Hybridoma supernatants were initially confirmed to exhibit ELISA positivity against the immunogen by ELISA assay and the EF5‐97.1 hybridoma was cloned by limiting dilution.

### 2.9 Fluorescence‐activated cell sorting (FACS)

Patients (*n* = 6) diagnosed with early onset preeclampsia (28–32 weeks gestation) and their gestation age matched normal controls (*n* = 6) were recruited and informed written consent was obtained from all recruits. The Oxfordshire Research Ethics Committee C approved this study (Ref. 07/H0606/148).

Venous blood samples in sodium citrated vacutainers (BD Biosciences, UK) were collected and transported to the laboratory within 30 min of sampling. To isolate platelet‐free plasma (PFP), vacutainers were first centrifuged at 1500 *g* at room temperature for 15 min in a swing bucket centrifuge (Beckman Coulter, UK). Plasma was collected and 550 μl were aliquot to Eppendorf tubes and further centrifuged for 2 min at 13,000 *g* in a fixed angle microfuge (Thermofisher, UK) at room temperature. The top 500 μl of PFP was transferred to a new Eppendorf tube and frozen down at –80°C for storage before flow analysis.

Mouse monoclonal anti‐ELTD1 antibody (clone 97.1; isotype IgG1) was conjugated to Allophycocyanin (APC) using a Lightening‐Link Antibody Labelling kit (Novus Bio‐Techne Ltd., UK) following manufacture's instruction. Mouse monoclonal anti‐placental alkaline phosphatase (PLAP) (isotype IgG1) is an in‐house antibody that was custom conjugated to R‐Phycoerythrin (R‐PE) by Biolegend. Both IgG1/APC and IgG1/PE isotype controls were from Biolegend (UK). Used CD41/FICT, CD31/PE and CD235/PE Vio770 (used to label platelet, endothelium and erythrocyte markers respectively) were all recombinant engineered antibodies (REAs) and their respective REA controls were from Miltenyi Biotech, UK. All antibodies were filtered using 0.1 μm filter unit (Millipore, UK) to remove aggregates before use. Ten microlitres of Fc receptor blocker was added to all staining conditions for 10 min prior to staining. Antibodies were titrated to obtain as bright as possible signals with the brightest events below 10^5^ on the fluorescence intensity scale, to ensure fluorescence signals being within linearity range.

PFP sample staining was conducted as previously published. Briefly, 100 μl of thawed PFP was incubated with anti‐ELTD1/APC (1 μg/ml), anti‐CD41/FITC, anti‐CD31/PE and anti‐CD235/PE Vio770 (2 μl of each antibody per sample as per supplier instructions). Samples were incubated at room temperature for 15 min in the dark before analysis. Controls included non‐stained samples (no fluorochromes), isotype controls for ELTD1 or CD31, and antibody cocktails. After sample acquisition, stained samples were treated with 1% NP‐40 detergent and analysed once more with a flow cytometer.

Comparison of CD31 and ELTD1 expression in cell culture supernatants was performed by staining 100 μl of sample after it had been centrifuged twice at 1500 *g* to remove cell debris. Non‐stained, single ELTD1 or CD31 stained samples or antibody cocktail were used as controls. Events above 1% of antibody cocktail, which gave the highest background signals, were considered positive.

A BD LSR‐II was used to analyse all samples as previously described. Briefly, Cytometer Setup and Tracking (CS&T) beads were run on each experimental day. Photomultiplier Tubes’ Voltages (PMTVs) from CS&T beads run with exception to SSC was applied to all experiments. Both SSC and FSC parameters were set to log scale to maximise the light scatter display. PMTV for the SSC, which determines machine's light scatter resolution, was set to be able to detect beads sizes above the 0.59 mm silica beads in Apogee beads (Apogee, UK). No thresholds other than an arbitrary threshold of 200 was applied to SSC. Machine flow rate was set to 10μl/minute using Trucount beads. This was set at the beginning and checked after acquiring the last sample at the end of each experiment. Before sample analysis, machine's basal noise, which is indication of machine's cleanness, at < 8 counts/second and 1000 counts as a total count in 2 min was obtained. Machine's basal noise is defined as a reproducible count from running a 0.1 μm filtered PBS at 10 μl/min at above specified SSC PMTV and threshold.

Stained samples were initially analysed at 1 in 5 dilutions made with 0.1 μm filtered PBS. Depending on the event rate (counts/second) obtained from this initial diluted sample, appropriate further sample dilutions were made so that event rate was less than 1000 counts/second. Sample dilution factors necessary to achieve 1000 counts/second were between 20 and 100 for all samples tested. In addition, CD41+ and CD235a+ counts from these serial titrated samples were compared, to confirm counts of CD41+ and CD235a+ events in the samples analysed showing a reduction correspondent to the titration factor. All samples were acquired for 2 min.

Sample analysis was performed using FlowJo software (Version 10, BD Biosciences, UK). Recorded events from a light scatter range similar to 0.59–1.3 μm silica beads were gated and events from this gate were analysed first by expression of CD235 and CD41. Events double negative for CD235 and CD41 (markers of erythrocytes and platelets respectively) were further analysed for expression of ELTDI and CD31. The concentration of CD235^–^CD41^–^CD31^+^ELTD1^+^ events as counts/ml in the original samples was calculated following this formula: [(CD235^–^CD41^–^CD31^+^ELTD1^+^ counts in 2 min run)/20 μl] x dilution factor x 1000. Statistic difference of events which were CD235^–^CD41^–^CD31^+^ELTD1^+^ (counts/ml) were compared between patients and controls by paired *t*‐test.

### 2.10 Xenograft mouse models

All animal procedures were carried out in accordance with the UK Home Office Licence 30/3197. Total 100 μl of Matrigel (BD Biosciences) containing 1 × 10^7^ MDA‐MB‐231 cells, were inoculated into the mammary fat pad (MFP) of female BALB/c severe combined immunodeficiency (SCID) mice (Charles Rivers) aged 5–6 weeks. At day 9, when tumours were palpable, 100 μl (1 × 10^9^ EVs/ml) of EVs derived from HMEC‐1 endothelial cells resuspended in PBS were injected around the tumour site at equal spacing and rotation. Injections were carried out three times a week for 3 weeks (nine injections in total). Tumours were measured by calliper at least three times a week.

### 2.11 Immunohistochemistry (IHC)

IHC analysis for cell proliferation (Ki67; Qigent), microvessel density (Endomucin; Abcam), hypoxia (CA9; Abcam). Slides were dewaxed and antigen retrieval performed in pH 6 buffer. Endogenous peroxidase activity was blocked before slides were stained for 1 h at room temperature. Slides were stained using the FLEX staining kit (Agilent) and visualised using 3,3′‐Diaminobenzidine (Flex‐DAB) chromogen and counterstained with haematoxylin.

Expression of markers and viable/necrotic areas were quantified on whole sections quantitatively by using the Visiopharm Integrator System. HDAB‐DAB colour deconvolution band was used to detect positively stained cells. Appropriate thresholds levels were checked against control xenografts staining before being set and the xenografts from all groups were then analysed.

### 2.12 Quantitative real‐time PCR (QPCR)

RNA was extracted using TRI Reagent® (Sigma) according to manufacturer's instructions and reverse transcription was performed using the High Capacity cDNA Archive Kit (Applied Biosystems). qPCR reactions were set up using SensiMix SYBR (Bioline) with 20 ng of cDNA and 0.3 μM of each oligonucleotide. The qPCRs were run in a RotorGene Q (QIAGEN) and the cycling conditions used were: 95°C for 10 min followed by 40 cycles of 95°C for 15 s and 60°C for 60 s. Primers used are listed in Table S1.

### 2.13 Extracellular vesicle (EV) isolation

HUVEC, HMEC‐1 and MDA‐MB‐231 cells were grown in media containing Exosome‐Depleted FBS (Gibco) for 48 h to obtain EVs. EVs were harvested from cell media by ultracentrifugation as described previously (Théry et al., 2006). The cell supernatants were harvested and cells removed by centrifugation at 450 *g* for 5 min at 4°C. The large vesicles and cell debris were then removed by centrifugation at 2000 *g* for 20 min at 4°C. The EVs were then isolated by ultracentrifugation in an Optima XPN ultracentrifuge (Beckman) for 2 h at 110,000 *g*. The pellet was washed in cold PBS and centrifuged again before resuspension in PBS or RIPA buffer (Sigma).

### 2.14 Protein identification by mass spectrometry

EVs were precipitated and desalted with chloroform/methanol (Wessel & Flügge, 1984). Samples were resuspended in buffer that contained 6 M urea and 100 mM Tris pH 7.8, followed by reduction, alkylation, and in‐solution trypsin digestion as described previously (Fischer & Kessler, 2015). Briefly, samples were subjected to liquid chromatographic separation on a nEASY column (PepMAP C18, 75 μm x 500 mm, 2 μm particle, Thermo Fisher) with a flow rate of 250nl/min and a linear gradient of 2%–35% Acetonitrile in 0.1% formic acid/5% DMSO over 60 min. Survey scans were acquired as a resolution of 70,000 and the most abundant 15 precursors were selected for HCD fragmentation at 28% normalized collision energy. MS/MS spectra were acquired at a resolution of 17,500 for up to 128 ms. Selected precursors were excluded for 27 s.

Label free quantitation was conducted in Progenesis QI (Waters) after importing protein identifications from Mascot 2.5 (Matrix Science) using standard settings. The mass spectrometry proteomics data have been deposited to the ProteomeXchange Consortium via the PRIDE (Perez‐Riverol et al., 2022) partner repository with the dataset identifier (PXD_tbc).

### 2.15 NanoSight analysis of EV size

EV preparations were assessed using the NanoSight NS300 (Malvern Panalytical). The machine utilises Nanoparticle Tracking Analysis (NTA) of light scattering and Brownian motion in order to obtain the size and distribution of samples in liquid suspension.

EV preparations were stored at −80°C, thawed at room temperature, and then diluted in phosphate‐buffered saline (PBS) that had been shown to be free of any contaminant particles. The culture media from all cell types were analysed at the same dilution, independent of the sample, and no contaminating particles were detected.

### 2.16 Statistical analysis

Prism 8 (Graphpad Software) was used to analyse the results and data are shown as mean ± standard deviation (SD). Student's *t*‐test was used to compare two unpaired groups. Significance is denoted as: *p ≤ 0.05, **p ≤ 0.01, ***p ≤ 0.001

## 3 RESULTS

### 3.1 ELTD1 is incorporated into endothelial EVs

**FIGURE 1:**
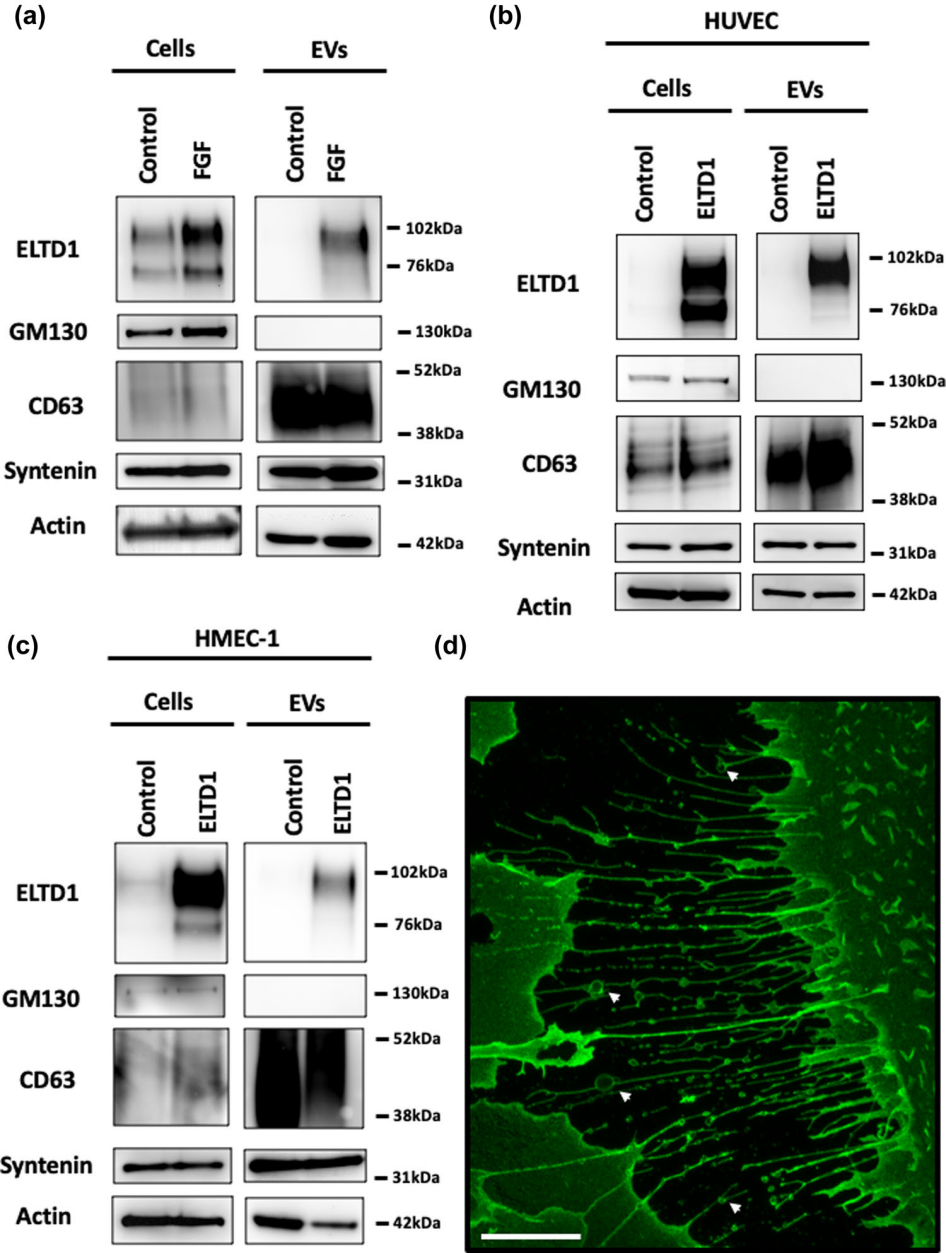
ELTD1 is incorporated into endothelial EVs. (a) Western blot of HUVEC cells and isolated EVs after treatment with 10 ng/ml FGF for 48 h. (b) Western blot of HUVEC cells and (c) HMEC‐1 cells and their associated EVs after increased expression of ELTD1. (d) Image of HUVEC expressing HA‐tagged ELTD1 stained with anti‐HA‐Alexa Fluor 488. Image was taken at 63x magnification. Migrasomes indicated by arrows. Scale bar = 20μm

We have previously shown that cellular ELTD1 levels are increased after treatment with fibroblast growth factor (FGF) (Masiero et al., 2013). We treated HUVEC with 10 ng/ml FGF for 48 h to see if upregulated ELTD1 is incorporated into EVs. As previously observed (Masiero et al., 2013), FGF increased the level of ELTD1 in total cell lysates (Figure 1a). The extracellular domain (ECD) of ELTD1 undergoes N‐glycosylation and this is detected as two bands at approximately 95 and 70 kDa (Masiero et al., 2013). ELTD1 was also present in the EVs but only the mostly highly glycosylated form was identified and this increased upon FGF treatment (Figure 1a). To further investigate the effect of ELTD1 incorporation into EVs, we increased the level of ELTD1 in HUVEC and HMEC endothelial cell lines by infection with full length ELTD1‐coding lentivirus or virus control at MOI 5 (Figure 1b,c). This method is frequently used in the GPCR field when attempting to elucidate the signalling pathways of receptors with unknown ligands as in the ELTD1 case (Gupte et al., 2012). Increasing the expression in both cell types also resulted in ELTD1 incorporation into EVs. The vesicles contained proteins that are normally present in EVs (CD63 and syntenin) and did not contain GM130, which is present in the Golgi, confirming EV purity (Lötvall et al., 2014) (Figure 1a–c). FGF treatment or ELTD1 overexpression did not affect vesicle number or size as assessed by Nanoparticle Tracking Analysis (NTA) using the NanoSight NS300 (Figure S1A, S1B and S1C). Recently, another type of EV has been described called ‘migrasomes’. They form at the ends or intersections of retraction fibres deposited by migrating cells and they release EVs when they rupture (Ma et al., 2015). We have previously shown that the ECD of ELTD1 is deposited on cell tracks (Sheldon et al., 2021) and ELTD1 is associated with migrasome‐like structures (Figure 1d).

### 3.2 EVs with higher levels of ELTD1 are pro‐angiogenic

**FIGURE 2:**
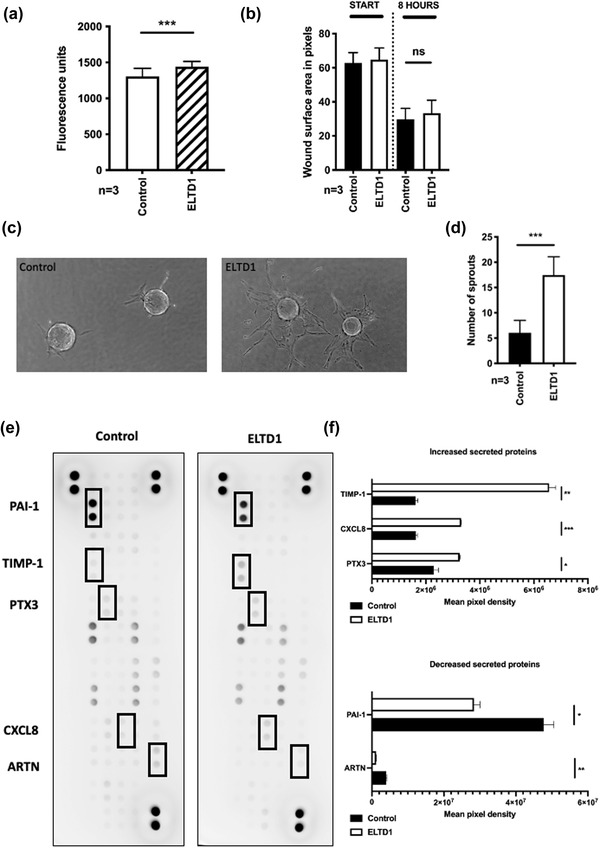
EVs with higher levels of ELTD1 are pro‐angiogenic. (a) Proliferation of HUVEC after 72 h of EV treatment. Cells were seeded at 1000 cells/96 well and quantified using the CYQUANT cell proliferation assay (ThermoFisher) (b) Migration of HUVEC after 48 h of EV treatment. Migration was assessed using the IncuCyte® live cell analysis system (Sartorius). Endothelial cells were grown to confluence in a 24‐well ImageLock plate and a scratch wound was made using the IncuCyte® wound maker (Sartorius). Images were collected every hour until the wound had closed and the images were analysed using Fiji software. (c) Representative images of endothelial sprouting 48 h after EV treatment. (d) The number of sprouts were counted after 48 h and quantified. (e) Angiogenesis array using HUVEC lysates collected 24 h after EV treatment. HUVEC (5×10^5^) were incubated for 24 h with 1×10^9^/ml EVs (control and ELTD1 expressing) and then lysed in Lysis Buffer 17 (R&D Systems). 300μg of each was used in the ProteomeProfiler Angiogenesis Array (R&D Systems) (f) Quantification of the dot intensity using Fiji software. Data were analysed by the unpaired *t*‐test *p < 0.05; **p < 0.01; ***p < 0.001. *n* = 3 biological replicates

Control (Control‐EV) or ELTD1 enriched (ELTD1‐EV) HMEC‐1 EVs were added to HUVEC at 1×10^9^ vesicles/ml. ELTD1‐EVs slightly increased cell proliferation when compared to the control (Figure 2a) but they had no effect on cell migration (Figure 2b). They did however, have a much greater effect on endothelial sprouting (Figure 2c,d). To confirm the pro‐angiogenic effects of the ELTD1+ vesicles we used the angiogenesis inhibitor, Sunitinib (Hao & Sadek, 2016). Sunitinib is a multi‐targeted receptor tyrosine kinase inhibitor with high activity against the VEGFR2 and PDGFRΒ receptors which are involved in endothelial sprouting. Pre‐incubation with 1 μM Sunitinib inhibited sprouting after treatment with both control and ELTD1‐EVs but its effects were greater in ELTD1‐EV treated samples (Figure 2a and S2B). Lysates from cells exposed to EVs for 48 h were then analysed with an Angiogenesis Array. Five proteins were found to be differentially expressed in ELTD1‐EV treated cells when compared to the Control‐EV treated cells, with TIMP‐1, CXCL8 and PTX3 being upregulated and PAI‐1 and ARTN downregulated (Figure 2e and f).

### 3.3 ELTD1‐enriched EVs contain proteins related to haemostasis

**FIGURE 3:**
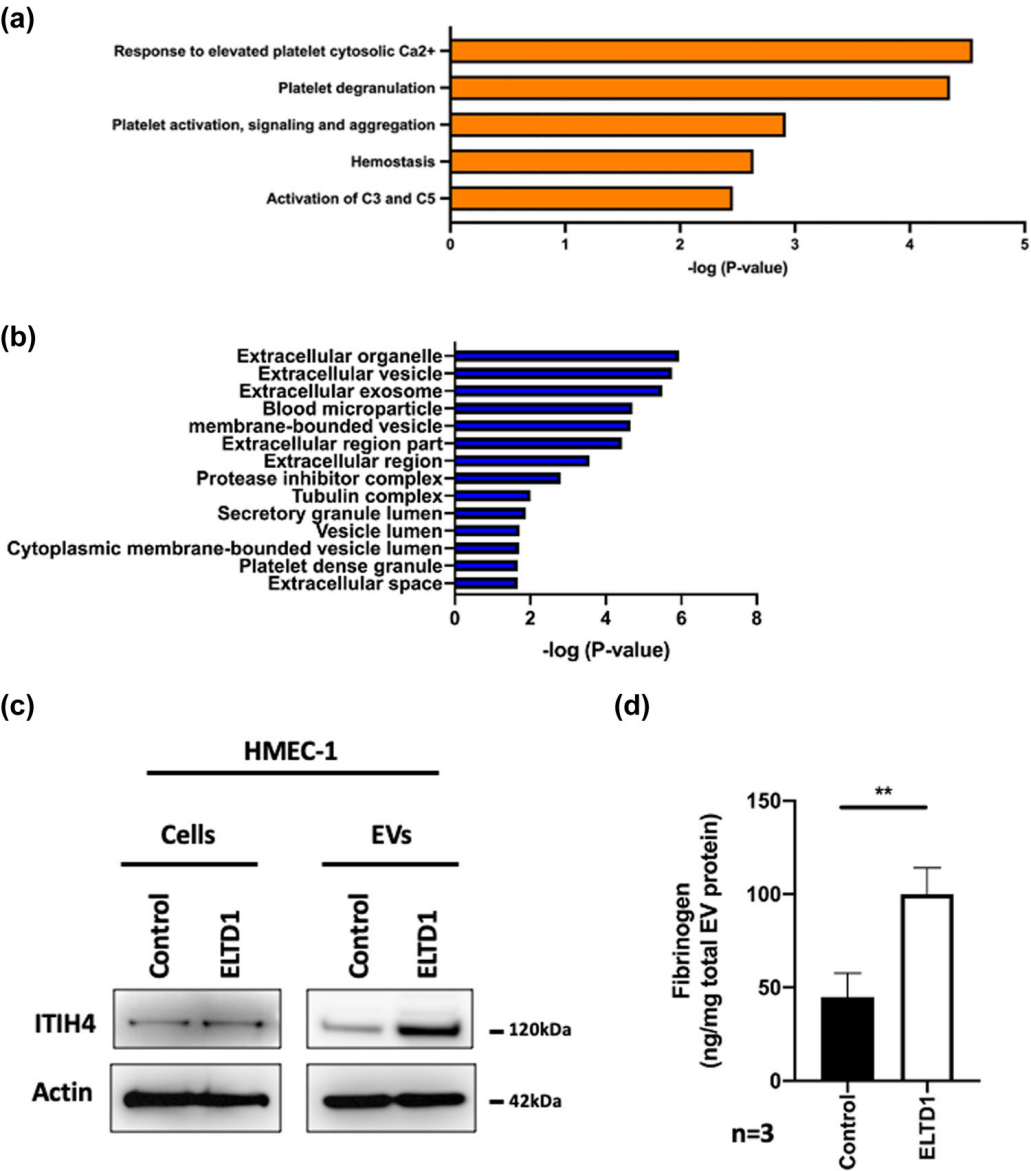
Proteome of endothelial EVs with higher levels of ELTD1. We performed mass spectrometry‐based proteomic analysis on three EVs batches from both control and ELTD1‐overexpressing HUVEC and HMEC‐1. HUVEC and HMEC‐1 data sets were pooled and significantly upregulated proteins common to both types of ELTD1‐EVs were identified (a) Enrichr (http://amp.pharm.mssm.edu/Enrichr/) Reactome pathway analysis of significantly upregulated proteins in the vesicles of endothelial cells with higher levels of ELTD1. (b) Compartments associated with the upregulated proteins (Jensen Compartment library, Enrichr). (c) Western blot confirming increased levels of ITIH4 in ELTD1 EVs. (d) ELISA confirming increased levels of fibrinogen in ELTD1 EVs. Three batches of control and ELTD1 enriched HMEC‐1 EVs were lysed in lysis buffer (CST) and 75 μg of total protein each used in triplicate. The Fibrinogen ELISA was performed as per manufacturer's instructions (Abcam). Data were analysed by the unpaired *t*‐test **p < 0.01. *n* = 3 biological replicates

We performed mass spectrometry‐based proteomic analysis on three EVs batches from both control and ELTD1‐overexpressing HUVEC and HMEC‐1. HUVEC and HMEC‐1 data sets were pooled and significantly upregulated proteins common to both types of ELTD1‐EVs were identified and listed in Table S2. Enrichr Reactome Pathway Analysis of these proteins identified significant upregulation of five biological processes: response to elevated platelet cytosolic Ca2^+^; platelet degranulation; platelet activation, signalling and aggregation; haemostasis and activation of C3 and C5 complement proteins (Figure 3a). The proteins identified also mapped to cellular components which are consistent with EVs such as extracellular organelle; EV and extracellular exosome (Figure 3b). The upregulation of two of the proteins in ELTD1‐EVs was confirmed either by Western blot (inter‐alpha‐trypsin inhibitor heavy chain H4, ITIH4) or ELISA (fibrinogen) (Figure 3c,d).

### 3.4 The ECD portion of ELTD1 is pro‐angiogenic when present in EVs

**FIGURE 4:**
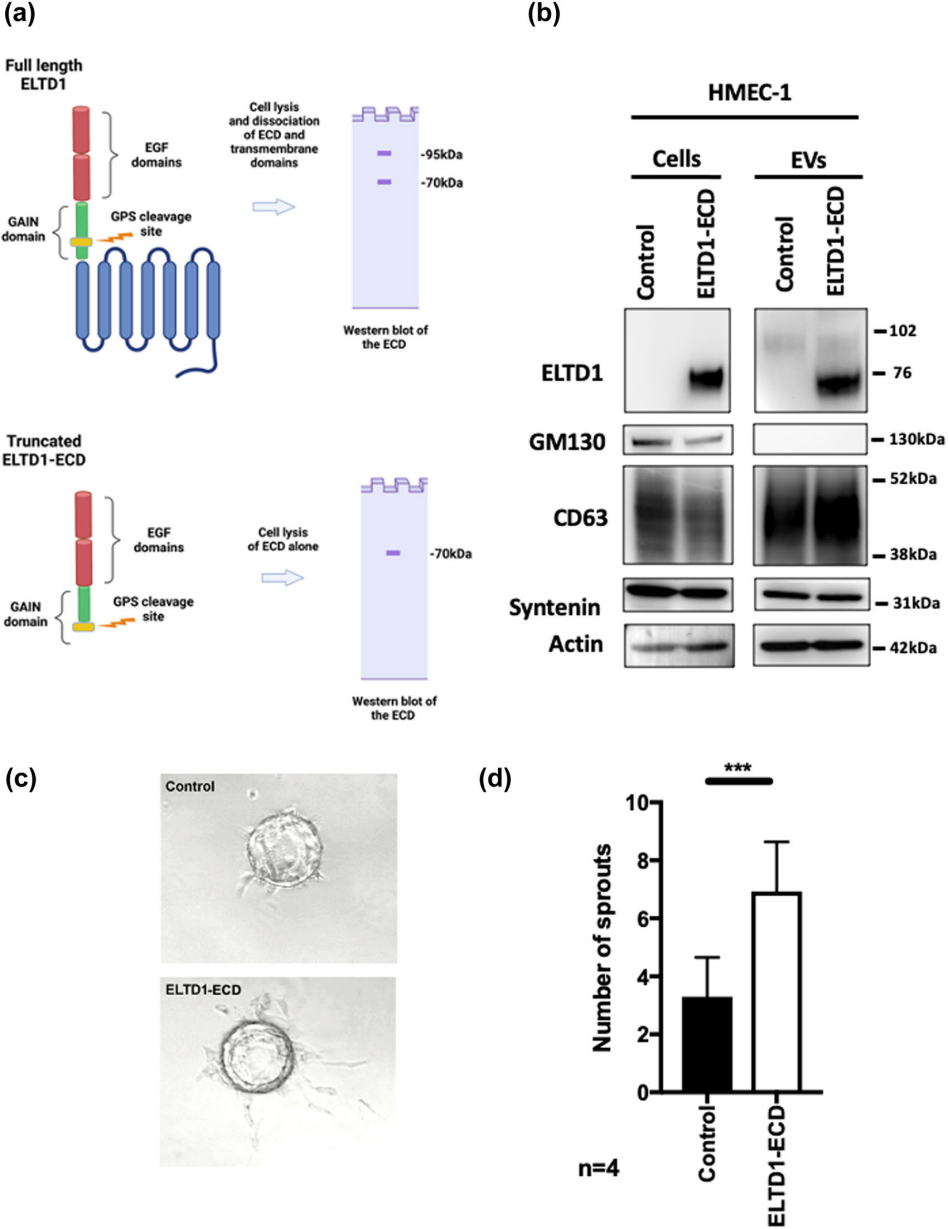
EVs containing the ECD of ELTD1 are also pro‐angiogenic. (a) ELTD1 construct design and western blot. Full length construct includes the two EGF domains, the GPCR‐Autoproteolysis Inducing (GAIN) domain with autocatalytic processing at the GPCR‐proteolytic site (GPS). After cell lysis the ECD dissociates from the transmembrane domain and only the ECD is detected by the antibody. The ECD is glycosylated and is detected as two bands on a western blot. The extracellular domain (ECD) construct is truncated at the GPS site, terminating at amino acid 406. After western blot mainly the smaller glycosylated band is detected (b) Western blot of HMEC‐1 ELTD1‐ECD cells and associated EVs (c) Representative images of endothelial sprouting 48 h after EV treatment. (d) Quantification of endothelial sprouting. EVs added at 1×10^9^/ml. Data were analysed by the unpaired *t*‐test ***p < 0.001. *n* = 4 biological replicates

Although ELTD1 was significantly enriched in ELTD1‐EVs, matched peptides only mapped to the ECD portion of the protein. ELTD1 undergoes autoproteolysis after translation and the ECD is non‐covalently bound at the cell surface as a dimer (Favara et al., 2014). The mass spectroscopy data suggests that the intracellular domain is not trafficked to the vesicles and that the ECD can shed and associated with EVs. To see if the ECD alone can influence endothelial sprouting, HMEC‐1 cells were infected with a viral vector coding for the ECD portion of ELTD1 (truncated at the GAIN domain where the cleavage occurs (Favara et al., 2014) (Figure 4a) and the resulting vesicles isolated. After cell lysis of the endogenous full‐length ELTD1, the ECD dissociates from the transmembrane domain as it is non‐covalently bound. The antibody used for western blot analysis only detects the ECD and two bands are detected that are higher than the predicted molecular weight as it is glycosylated (95 and 70 kDa). Contrary to what was observed with full‐length ELTD1 overexpression, recombinant ELTD1‐ECD was mainly present as the smaller glycosylated form (70 kDa band) and this was observed in both cell lysates and corresponding EVs (Figure 4b). Functionally, ELTD1‐ECD‐EVs also increased endothelial sprouting when compared to the Control‐EVs but the effect was not as great as the endogenous highly glycosylated form (Figure 4c,d).

### 3.5 ELTD1 is incorporated into the EVs of tumour cells

Although ELTD1 expression is normally restricted to the endothelium, it has also been detected in tumour cells in vivo (Kan et al., 2018; Li et al., 2019). Approximately 10% of breast tumours express ELTD1 and we have shown that this expression can increase tumour growth and induce vessel normalisation in animal models (Sheldon et al., 2021). We therefore analysed the vesicles released from MDA‐MB‐231 human breast tumour cells after infection with ELTD1‐coding and control lentivirus. ELTD1 was expressed in cell lysates and, like with endothelial cells, the 95 kDa more highly glycosylated band was the only form incorporated into EVs (Figure S3A). In an angiogenesis assay, ELTD1‐EVs isolated from MDA‐MB‐231 cells had no effect on sprout number when compared to the control but significantly increased sprout length (Figure S3B, S3C and S3D). Other angiogenic factors in tumour EVs may be obscuring any additional effects.

### 3.6 ELTD1‐EVs increase angiogenesis in an in vivo model

**FIGURE 5:**
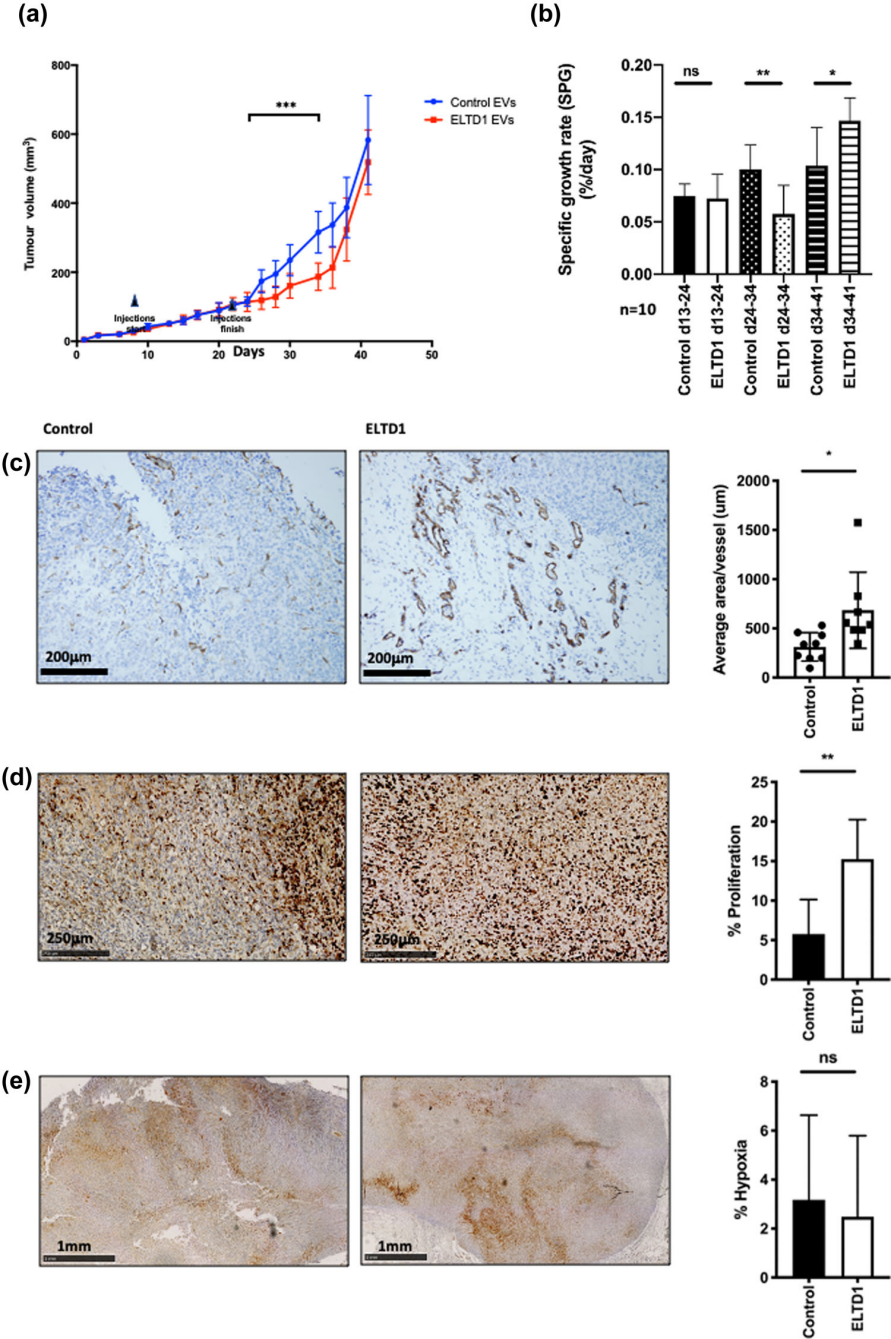
ELTD1 EVs influence tumour growth in vivo. (a) Xenograft tumour growth curves of MDA‐MB‐231 treated with 1×10^8^ control or ELTD1 enriched EVs isolated from HMEC‐1 endothelial cells (*n* = 10). (b) Quantification of specific growth rate (SPG) of the tumours during the experiment. Representative immunohistochemistry and quantification of (c) Endomucin vessel staining (d) Ki67 for cell proliferation (e) CA9 staining for hypoxia. *p < 0.05; **p < 0.01. *n* = 10 biological replicates

To assess the in vivo effects, we grew human MDA‐MB‐231 triple receptor negative breast cancer xenografts and injected them with 1×10^9^EVs/ml derived from HMEC‐1 endothelial cells and monitored tumour growth. EVs were injected between Day 9 and Day 24. During this period the specific growth rate (SPG) was the same between the two tumour growth curves (Figure 5a and 5B). After injections stopped the ELTD1‐EV injected tumours initially grew significantly more slowly (Day 24–34) but in the final stages of growth the ELTD1‐EV injected tumours grew significantly faster (Day 34–41), eventually catching up with the controls and reaching a similar size (Figure 5a,b). At this point the animals were sacrificed and the tumours embedded and sectioned for IHC analysis. Both vessel density (Figure 5c) and cellular proliferation were significantly increased in the ELTD1‐EV injected tumours (Figure 5d). The level of hypoxia was similar between the groups (Figure 5e).

### 3.7 EVs numbers increase during shear stress but ELTD1 incorporation into EVs is reduced

**FIGURE 6:**
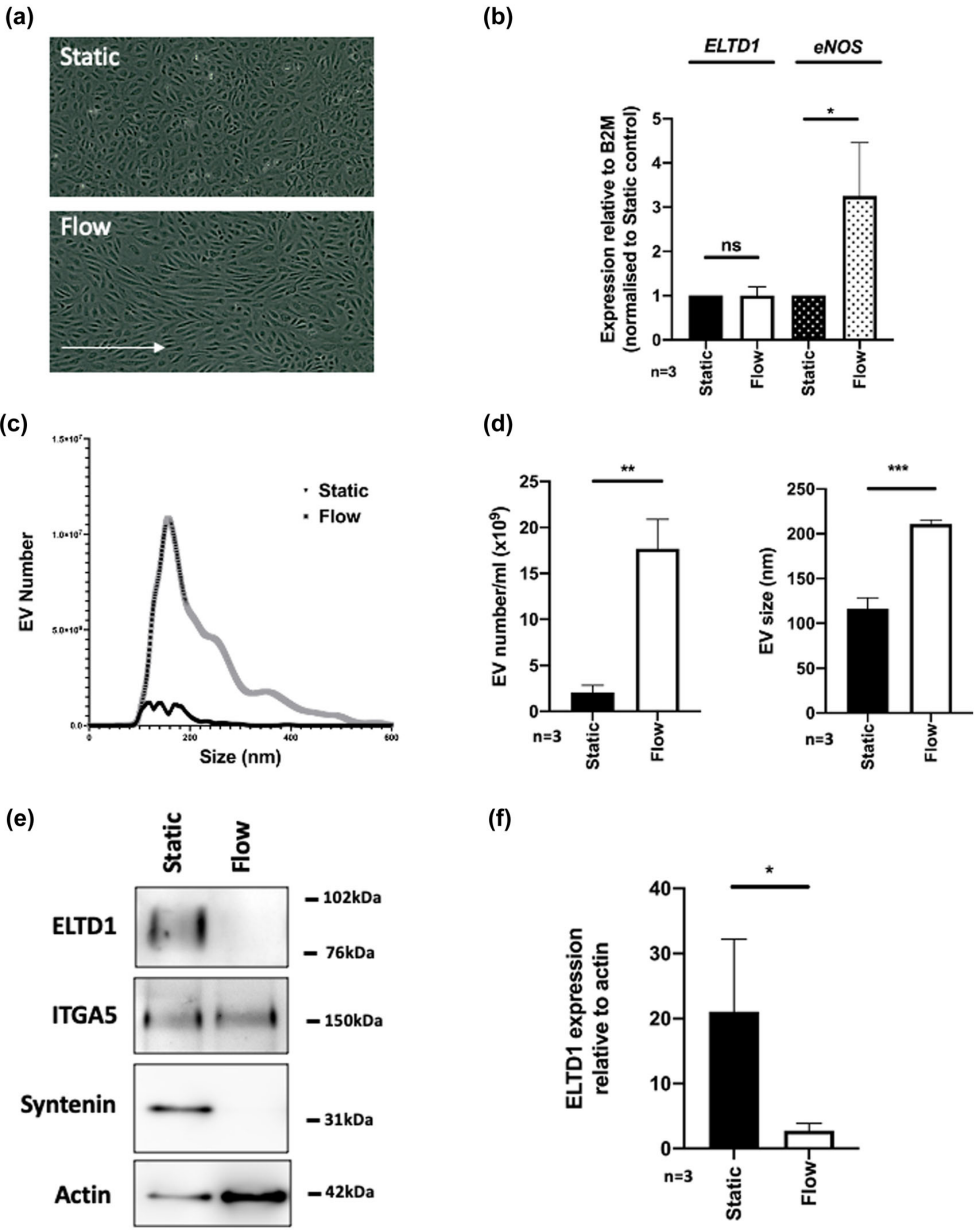
EVs numbers increase during laminar flow but ELTD1 incorporation into EVs is reduced. (a) Representative images of HUVEC in static conditions and under 15 dyn/cm^2^ laminar flow. HUVEC (7.5×10^4^) were seeded onto 0.4 mm microslides (μ‐Slide I Luer, ibidi) and used when they were fully confluent. Unidirectional flow of 15 dyn/cm^2^ was applied for 48 h using an ibid pump system housed in a 37°C tissue culture incubator. (b) QPCR of *ELTD1* and *eNOS* RNA levels in samples after 48hr static or flow culture. (c) Representative NTA data for static and flow samples. (d) Quantification of vesicle number and size using NTA (e) Western blot of EVs isolated after 48 h of static or flow culture (f) Quantification of ELTD1 incorporation into vesicles. Data were analysed by the unpaired *t*‐test *p < 0.05; **p < 0.01; ***p < 0.0001. *n* = 3 biological replicates

aGPCRs may be involved in sensing shear stress (Bassilana et al., 2019). As ELTD1 is expressed on the surface of endothelial cells it will be exposed to such forces in vessels. HUVEC were exposed to liquid flow and EVs isolated and compared to those isolated under static conditions. The cells were visualised after 48 h and a characteristic alignment was observed in the cells experiencing 15 dyn/cm^2^ laminar flow (Wang et al., 2013) (Figure 6a). QPCR was carried out on the cells at the time of EV harvest and the level of *ELTD1* was unaltered but the level of *eNOS*, which is a marker of shear stress (Boo & Jo, 2003), was increased (Figure 6b). NTA analysis of the supernatants revealed an increase in the number and size of vesicles released during flow when compared to the static control (Figure 6c,d). However the level of ELTD1 was significantly decreased in flow EVs (Figure 6e,f). Tetraspanins were not detected in the samples but the EV marker syntenin was decreased (Figure 6e). These data suggest that when endothelial cells are experiencing normal laminar flow and are quiescent, the ECD remains attached to the transmembrane domain and it is not detected in the EVs.

### 3.8 Detection of ELTD1^+^ EVs in cell supernatants

The proteomics data revealed that ELTD1‐EVs are enriched with proteins related to haemostasis therefore endothelial cells with activated ELTD1 may be experiencing vascular stress. ELTD1 incorporation into EVs could therefore potentially be used as a biomarker for vascular dysfunction. We developed a FACs procedure to identify ELTD1 in EVs present in cell supernatants and plasma. An antibody (97.1) was produced that recognises the extracellular EGF domain region of human ELTD1 and this was conjugated with PE for FACs experiments on cells, cell supernatants and plasma samples. Validation of the antibody is shown in Figure S4. FACs detected endogenous ELTD1 on HUVECs and this increased with overexpression (Figure S4A) and decreased when ELTD1 was silenced using ELTD1 siRNA (Figure S4B). Silencing was confirmed by Western blot (Figure S4B). EVs were isolated from the cell supernatants of control, ELTD1 overexpressing and silenced HUVEC and stained with PE‐labelled anti‐ELTD1 antibody in order to detect ELTD1 positive vesicles (Figure S4C and S4D). ELTD1 positive vesicles increased in the supernatant after ELTD1 overexpression and this signal could be eliminated when detergent was added to lyse the EVs (Figure S4C). ELTD1 knock‐down in EV producing cells also reduced ELTD1 positive EVs compared to the control siRNA (Figure S4D). These experiments confirmed that the generated anti‐ELTD1 antibody was also able to detect ELTD1 in EVs.

### 3.9 Analysis of plasma from pre‐eclampsia patients shows increased amounts of ELTD1^+^ EVs

**FIGURE 7:**
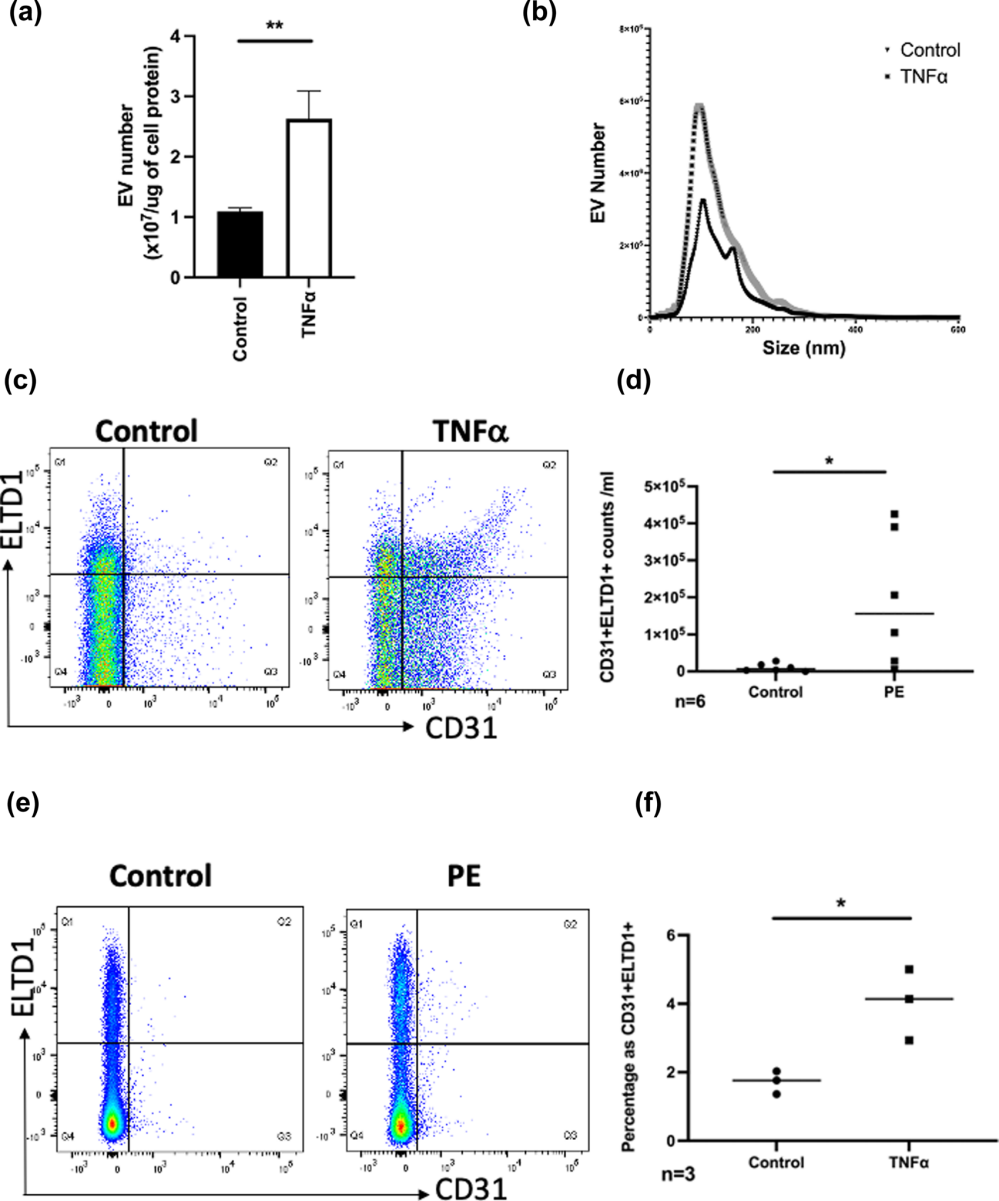
ELTD1 can be detected in cell supernatants and plasma samples. (a) Quantification of the number of EVs isolated from control and TNFα treated (10 ng/ml) HUVEC and (b) representative NTA image (c) Gating strategy and representative scatter plots of FACs detection of ELTD1 in EVs from control and TNFα treated HUVEC. (d) Quantification of CD31+ELTD1+ EVs in HUVEC supernatants. (e) Gating strategy and representative scatter plots of FACs detection of ELTD1 in EVs from control and PE patients plasma. (f) Quantification of ELTD1‐positive EVs in healthy and pre‐eclampsia plasma. Data were analysed by the unpaired *t*‐test *p < 0.05; **p < 0.01. *n* = 3 or 6 biological replicates

Pre‐eclampsia is a disease that results in severe maternal vessel dysfunction (Vanwijk, 2000). TNFα is a key player in the pathophysiology of pre‐eclampsia and can be used to induce a model of pre‐eclampsia in animals (Raghupathy, 2013; Sunderland et al., 2011). We therefore treated HUVEC with TNFα for 24 h and measured at the release of ELTD1 positive vesicles. After TNFα treatment, there was a significant increase in the total number vesicles and of EVs that were CD31+ single and CD31+ELTD1+ double positive (Figure 7a–d). The gating strategy is shown in Figure S5. To examine if an increase in ELTD1+CD31+ was also observed in a disease known to have vascular dysfunction we compared the number of CD31+ELTD1+ EVs between early onset pre‐eclampsia patients and gestational age matched normal controls. After excluding CD41+ platelets and CD235a+ erythrocyte EVs, ELTD1+CD31+ double positive EVs from the CD41‐CD235a‐ gate were greatly increased in the PFP collected from these patients compared to those from gestation age matched normal controls (18 fold increase, *p* < 0.05) (Figure 7e,f). The gating strategy is shown in Figure S6.

## 4 DISCUSSION

A number of GPCRs have been identified in EVs (Dores & Trejo, 2014). They are thought to be involved in EV biogenesis (Bebelman et al., 2020) and the receptors displayed on secreted vesicles can affect signalling on adjacent cells by acting as ligand scavengers (Shen et al., 2016). ELTD1 is readily incorporated into EVs but only the most highly N‐glycosylated form of the ECD is present. Glycosylated proteins are abundant in EVs and this glycosylation may enable sorting into vesicles (Carnino et al., 2020).

A defining feature of aGPCRs is that they are capable of auto‐proteolysis in their ECD creating an extracellular fragment and a seven‐transmembrane C‐terminal fragment (CTF) (Araç et al., 2012; Lin et al., 2010). There is increasing evidence to suggest that ECD detachment can result in receptor activation (Okajima et al., 2010; Paavola et al., 2011; Ward et al., 2011) and therefore the presence of ELTD1‐ECD in EV may reflect ELTD1 activation. A model has been proposed whereby the removal of the ECD exposes a Stachel peptide sequence (a cryptic tethered agonist), which then interacts with the extracellular loops of the transmembrane domain and initiates the conformational changes required to activate downstream signalling (Liebscher et al., 2014). A number of studies have also shown that the ECD can exist in a soluble form, for example, PKD1, ADGRL1, ADGRE5(CD97), GPR56, GPR126 and GPR116 (Abe et al., 2002; Fukuzawa & Hirose, 2006; Gray et al., 1996; Jin et al., 2007; Maser & Calvet, 2020; Silva et al., 2009). These can have independent functions, for example, the ECD of GPR126 is involved heart development and axon sorting (May et al., 2004), the ECD of GPR124 can promote endothelial cell adhesion and survival (Vallon & Essler, 2006) while the ECD of ADGRB1 inhibits endothelial cell migration and angiogenesis (Kaur et al., 2005).

ELTD1‐enriched endothelial EVs are pro‐angiogenic in vitro and in vivo. This effect could be inhibited by Sunitinib suggesting a role for VEGFR2 and/or PDGFRB in some of these processes. Indeed links between ELTD1 and the VEGF pathways have been previously reported. VEGF‐A can induce ELTD1 expression and anti‐ELTD1 antibodies can decrease VEGFR2 levels in mouse glioma models (Dieterich et al., 2012; Masiero et al., 2013; Ziegler et al., 2019). Expression of the ELTD1‐ECD alone also resulted in EVs that could increase endothelial sprouting but to a lesser extent than the EVs isolated from the full‐length ELTD1 expressing cells. This suggests that the ELTD1‐ECD protein has an active function separate from any Stachel‐like activation mechanism.

**FIGURE 8:**
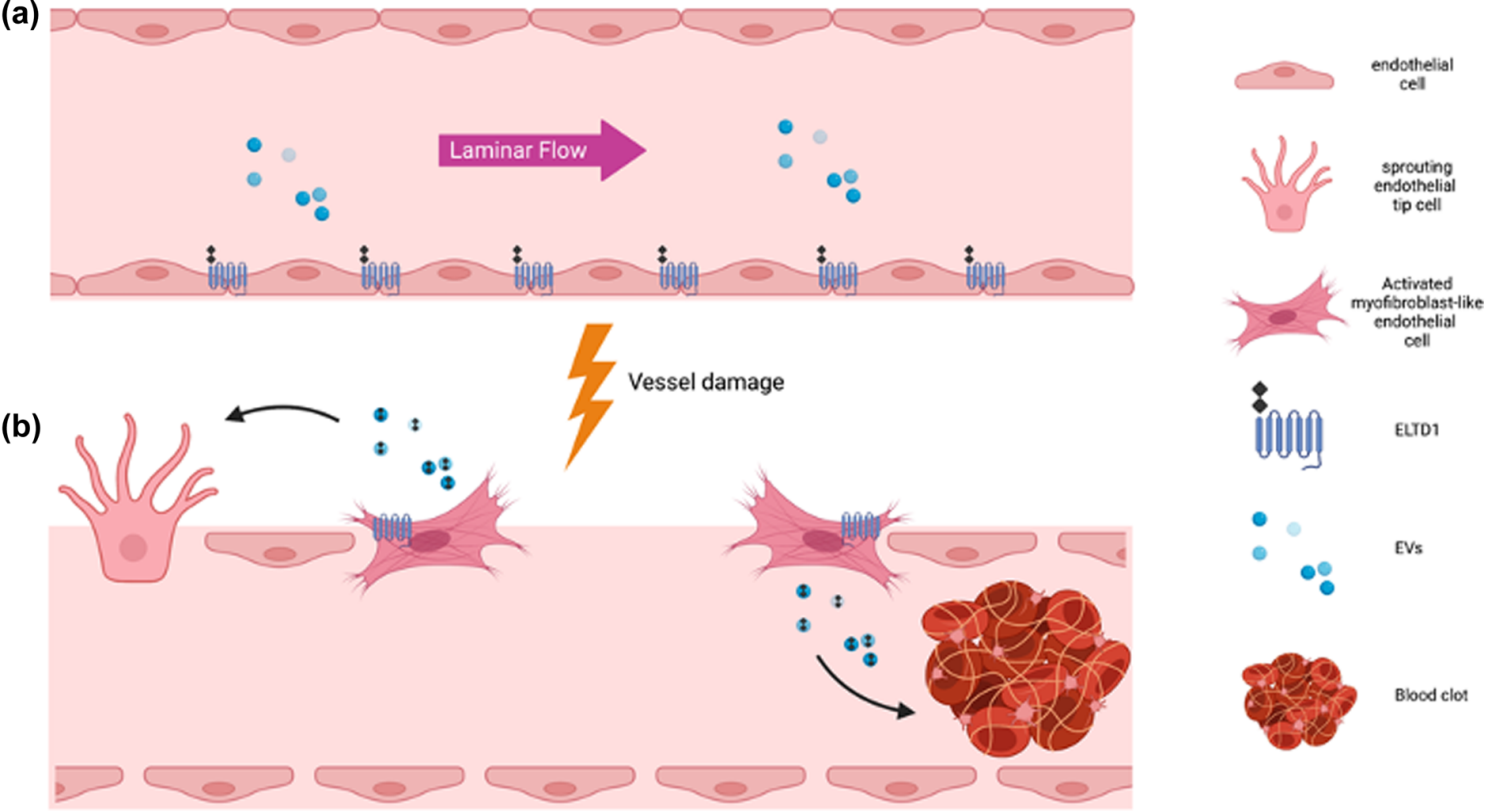
Summary of the role of ELTD1 and ELTD1+ EVs in vascular repair. (a) In normal vessels under laminar flow, ELTD1 is expressed at the cell membrane where it is inactive. The ECD remains tethered to the receptor and is not present in EVs (b) After vessel damage and loss of cell‐cell contact, the ECD of ELTD1 is removed and this activates the receptor. ELTD1 activation in endothelial cells results in a type II EndMT resulting in myofibroblast‐like cells that participate in vessel repair. These cells secrete EVs that contain the ECD of ELTD1 and they are pro‐angiogenic. The ELTD1+ EVs are enriched in proteins involved in haemostasis and may also be involved in this process

ELTD1+ vesicles were enriched with other proteins that can affect angiogenesis. There was an increase in those involved in haemostasis, particularly platelet activation, fibrin clot formation and regulation of the complement cascade (e.g., fibrinogen, inter‐alpha‐trypsin inhibitor heavy chains and complement C4A and C5). The co‐expression of ELTD1 with these types of proteins suggests a role for ELTD1 in haemostasis. There are four stages that occur during wound healing: haemostasis, inflammation, remodelling and proliferation (Reinke & Sorg, 2012; Wilkinson & Hardman, 2020). EVs have been previously implicated in tissue repair and are secreted from all human cell types where they can induce cell proliferation, promote vessel integrity and modulate the immune system (Roefs et al., 2020). Myofibroblasts play an important role in this process and activation of ELTD1 in endothelial cells results in the formation of myofibroblast‐like cells (Sheldon et al., 2021). These cells are more angiogenic and secrete factors that would influence the immune system and secrete ECM proteins which are required to restore the tissue after injury (Sheldon et al., 2021). It is now clear that they can also release EVs that can enhance the healing process. A summary of the potential involvement of ELTD1 in vessel repair is shown in Figure 8.

In the later stages of wound repair, the vessels are stabilized via recruitment of pericytes and smooth muscle cells, then blood flow can be initiated. Under flow endothelial cells become quiescent and anti‐thrombotic (Traub & Berk, 1998). *ELTD1* RNA levels are unchanged during laminar flow but ELTD1 protein incorporation into EVs was reduced, even though the number of vesicles increased. The size of the vesicles also changed and they had a reduction in EV markers. They still contained integrins suggesting vesicles produced during flow may be derived more from cell budding rather than endocytosis, therefore a reduction in vesicular ELTD1 may indicate a return to an inactivated state as the integrity of the vessel is restored. Some aGPCRs have been described as mechanosensors and use their large ECD to interact with their environment (Givens & Tzima, 2016; Petersen et al., 2015; Scholz et al., 2015). By a similar mechanism, ELTD1 may be able to sense vessel integrity and be activated, releasing its ECD, in response to vascular damage/dysfunction.

Tumours have been described as “wounds that never heal” (Dvorak, 1986). In a similar way to the proliferation phase of wound repair, they provide signals that are pro‐angiogenic and immune‐suppressive. However, tumour angiogenesis is poorly co‐ordinated and the resulting vessels are tortuous and leaky (Nagy et al., 2009). ELTD1 is upregulated in the vasculature of many tumour types (Masiero et al., 2013) which would be expected if it has a role in vascular “wound repair reaction”. Injection of ELTD1‐EVs into tumours during growth initially resulted in a reduction in tumour growth compared to the controls. ELTD1‐EV have higher levels of proteins involved in haemostasis, such as fibrinogen and therefore may initially restrict blood flow and ultimately tumour growth. Haemostasis occurs immediately but angiogenesis does not initiate until at least three days after wounding at which point haemostasis has been completed (Thompson et al., 1991). In line with this phenomenon, at later stages tumour growth accelerated, growing 46% faster than the controls to eventually reach a similar size. The vessels were larger at end‐point in the tumours injected with ELTD1‐EVs compared to the controls and this may be mediated by the pro‐angiogenic activity of the ELTD1‐enriched EVs and explain the enhanced proliferation.

HUVEC incubated with these vesicles had increased levels of CXCL8 and Pentraxin‐3 (PTX3) which have an important role in wound repair and positively regulate the immune response and angiogenesis (Heidemann et al., 2003; Presta et al., 2007; Ridiandries et al., 2018). In contrast TIMP‐1 negatively regulates inflammation and is anti‐angiogenic, but it does control ECM remodelling around wound healing (Amour et al., 1998; Gill et al., 2003; Qi & Anand‐Apte, 2015). PAI‐1 is a serine protease inhibitor that inhibits the breakdown of blood clots (Binder et al., 2002). Its levels were reduced in HUVEC after ELTD‐EV incubation and this reduction could help resolve the clot to allow angiogenesis to occur (Isogai et al., 2001; Wu et al., 2015). Artemin (ARTN) levels were also reduced. It has been described as having a role in angiogenesis and tumour growth but it has no published links to wound repair (Banerjee et al., 2012; Kang et al., 2009).

The incorporation of ELTD1 into EVs is not restricted to endothelial cells. Tumour cells expressing ELTD1 also produce ELTD1+ EVs and these are also pro‐angiogenic. Expression of ELTD1 in tumour cells resulted in EVs that were also capable of increasing endothelial sprout length but not number. The composition of EVs varies substantially from cell line to cell line and between cell types. Thus, the final effect of any protein will depend on other factors co‐expressed and this may differ from the cancer cell to the endothelial cells. Endothelial sprouting is regulated by VEGFR signalling but elongation of the stalk is regulated by Notch signalling (Jakobsson et al., 2010). The ECD of ELTD1 may also be capable of affecting this pathway as injection of ELTD1 enriched EVs into tumours increased vessel size, a phenotype that is also observed after Notch activation (Li et al., 2007). Interplay with the Notch pathway has been previously reported; Dll4 induced Notch signalling supresses the expression of ELTD1 in endothelial cells and inhibition of the pathway induces endothelial expression in vitro and in vivo (Del Toro et al., 2010; Masiero et al., 2013)

ELTD1 cancer cell expression has been identified in 35% of human breast tumours with 9% having high expression (Sheldon et al., 2021). Expression of Eltd1 in murine breast tumour cells resulted in increased tumour growth and metastasis. The vessels were also larger and better perfused suggesting a role for ELTD1 in tumour vessel normalisation (Sheldon et al., 2021). ELTD1‐enriched EVs produced by the tumour cells may also be involved in this mechanism.

The EVs released by ELTD1 expressing cells contain exosome markers but on average they are slightly larger (approximately 140 nm) than the estimated size of exosomes (50–100 nm). It is likely that some of the EVs released are being shed from the membrane. Migrasomes are a new type of EV identified in migrating cells and we detected the presence of ELTD1 in migrasome‐like structures by confocal microscopy. They are deposited on retraction fibres and they release vesicles that can be taken up by neighbouring cells (Ma et al., 2015). Cell migration plays an important role in vascular biology and migrasomes have been implicated in vascular homeostasis and vessel repair (Zhang et al., 2020) therefore some of the ELTD1+ vesicles may be derived from migrasomes.

EVs have gained substantial interest as potential biomarkers as they are detectable in blood and other biological fluids. Their content reflects the characteristics of the parental cell and they can be dynamically monitored (Lane et al., 2018). ELTD1 has been previously detected in the plasma of breast cancer patients using Liquid Chromatography‐Electrospray Ionization‐Mass Spectroscopy (LC‐ESI‐MS) (Dufresne et al., 2019). Since ELTD1 is involved in vessel biology and is detectable in vesicles when ELTD1 levels are elevated and presumably active then it has the potential to be a biomarker of vessel dysfunction.

FACs has the advantage that it can quantify vesicles from a wide variety of biological fluids however small EVs are hard to detect as they scatter little light and can be masked by artefacts such as doublets, aggregates, or swarm effects (Libregts et al., 2018). Conventional flow analysis can detect smaller particles (>250 nm) if the signal is strong enough, particles of this size constitute approximately 10% of the vesicles produced by HUVEC. Although this is only a small proportion of the vesicles present in the cell supernatant, we could clearly detect ELTD1 and this increased when ELTD1 was overexpressed.

Pre‐eclampsia is a pregnancy‐specific disorder that leads to generalized maternal vessel dysfunction (Vanwijk, 2000). Large increases in EVs occur early in pre‐eclampsia and early indications of functional severity would facilitate therapy to prevent foetal loss and maternal risk (Palma et al., 2021). FACs has been previously used to detect proteins present in EVs that are associated with this disease such as neprilysin and placental alkaline phosphatase (PLAP) (Dragovic et al., 2015; Gill et al., 2019). Interestingly, we found an > 18 fold increase in CD31+ELTD1+ vesicles in the plasma of patients with pre‐eclampsia compared to healthy controls. We speculate that the ELTD1+ EVs are released in response to vessel dysfunction and that these vesicles may be able to help repair vessels which are widely affected in this condition, as we have found they are pro‐angiogenic in vitro and in vivo. Therefore an increase in ELTD1+EVs in the plasma is a potential biomarker of poor vessel integrity and damage.

In summary, we have shown a functional role for the ECD of ELTD1 and a potential clinical relevance of ELTD1 in endothelial derived‐EVs. These vesicles may have a role in pathophysiology and the presence of ELTD1 could be used as a biomarker of vessel integrity not only in pre‐eclampsia, but in other diseases where vascular dysfunction plays a role.

## AUTHOR CONTRIBUTIONS

Helen Sheldon, paper concept and design, performed experiments, analysed data, prepared figures, wrote paper; Wei Zhang, Esther Bridges, performed experiments, analysed data, prepared figures. Koon Hwee Ang, Demin Li, Salwa Lin performed experiments. Pat Whiteman, Penny A. Handford and Demin Li produced and validated the anti‐ELTD1 antibodies; Massimo Masiero and Roman Fischer, performed experiments and analysed data. Manu Vatish, Alison H. Banham and Adrian L. Harris supervised research and provided critical revision of paper.

## CONFLICTS OF INTEREST

None.

## Supporting information

Supporting Information

Supporting Information

Supporting Information

Supporting Information

Supporting Information

Supporting Information

Supporting Information

Supporting Information
