# Supplementary figures and images for: ELTD1 is present in extracellular vesicles derived from endothelial cells as a cleaved extracellular domain which induces in vivo angiogenesis
Source: J Extracell Biol. 2022 Aug 2;1(8):e52. doi: 10.1002/jex2.52 (PMC11080856; doi:10.1002/jex2.52)

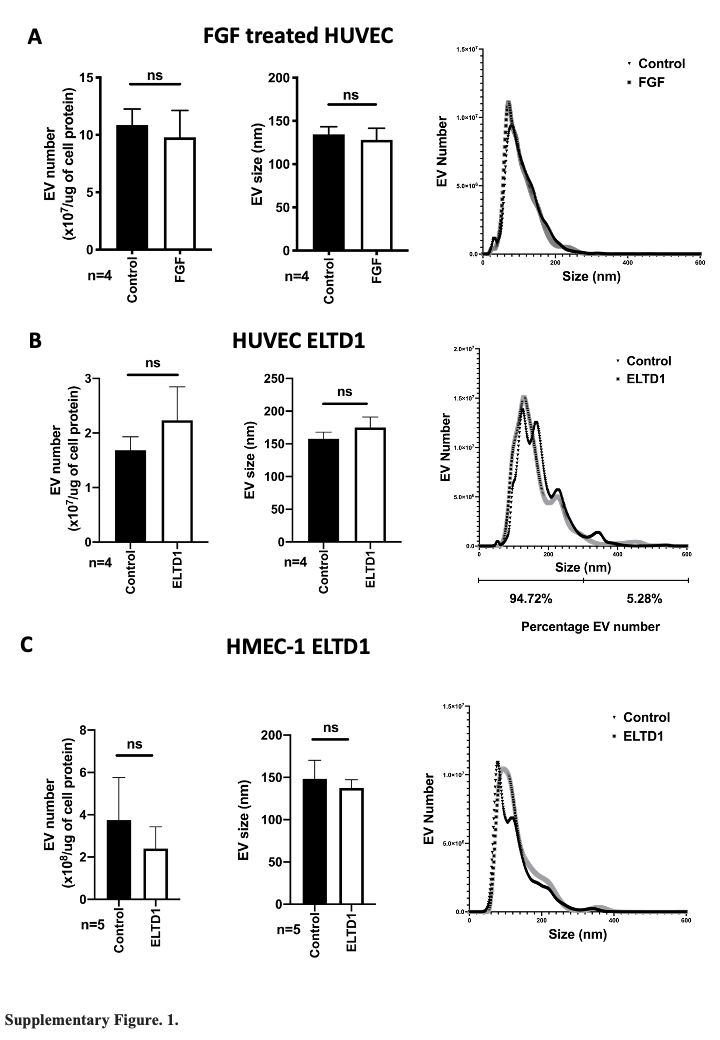

Supplement: Supplementary file 1 — Supporting Information [file JEX2-1-e52-s003.tiff]

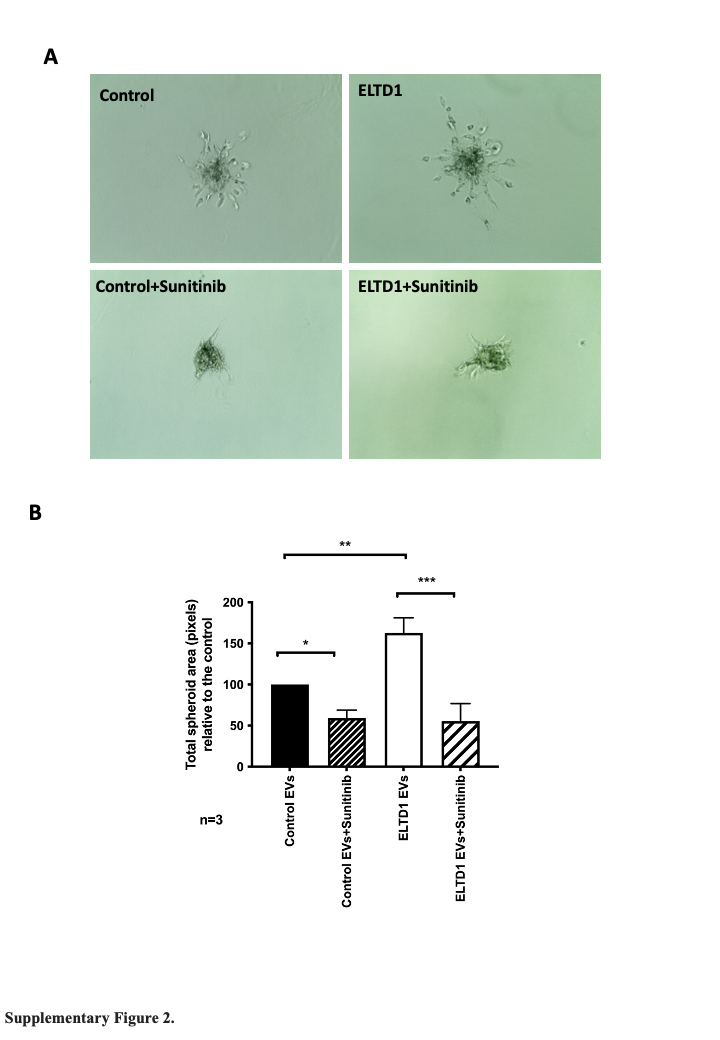

Supplement: Supplementary file 2 — Supporting Information [file JEX2-1-e52-s006.tiff]

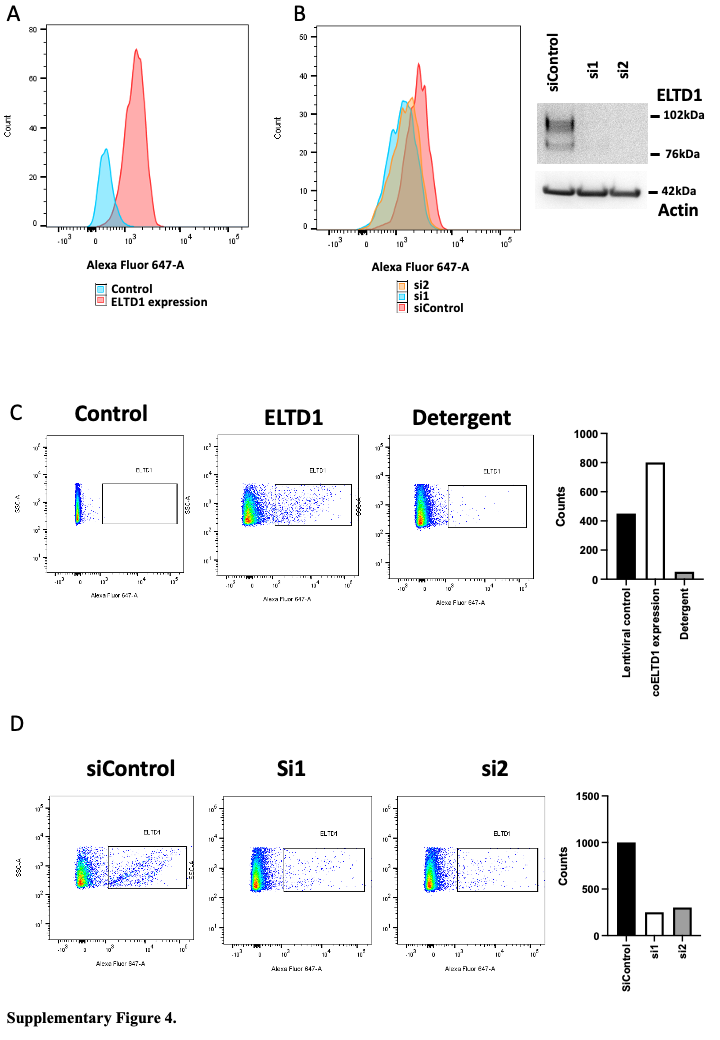

Supplement: Supplementary file 4 — Supporting Information [file JEX2-1-e52-s008.tiff]

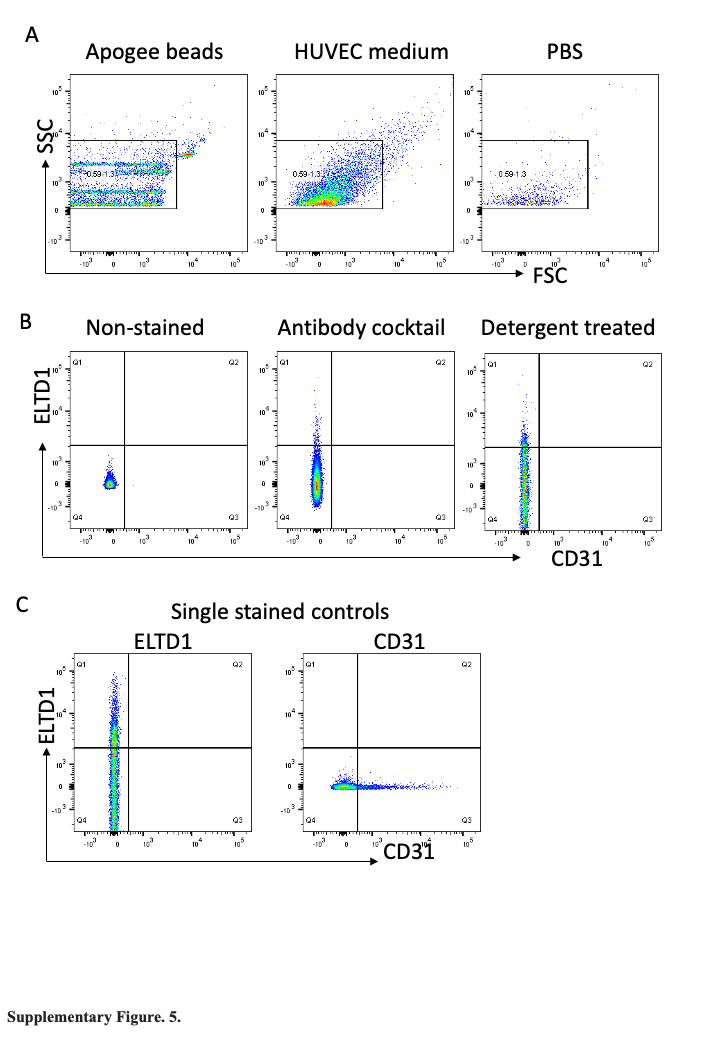

Supplement: Supplementary file 5 — Supporting Information [file JEX2-1-e52-s005.tiff]

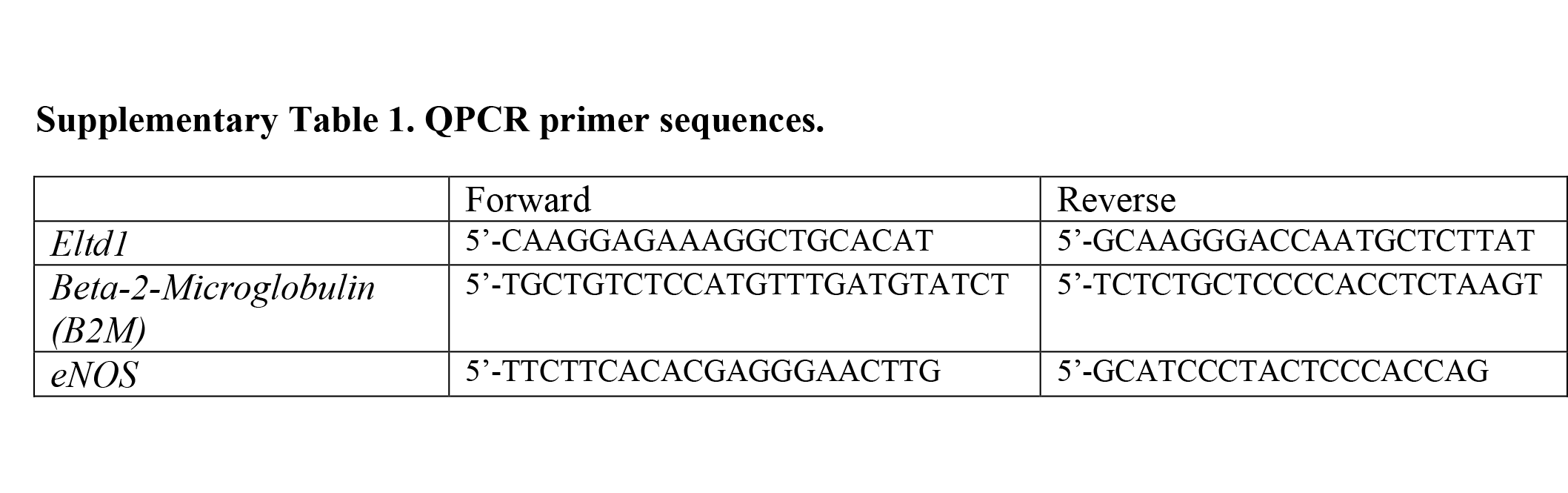

Supplement: Supplementary file 7 — Supporting Information [file JEX2-1-e52-s004.tif]
